# Abundance and dynamics of anopheline larvae in a highland malarious area of south-central Ethiopia

**DOI:** 10.1186/1756-3305-5-117

**Published:** 2012-06-13

**Authors:** Abebe Animut, Teshome Gebre-Michael, Meshesha Balkew, Bernt Lindtjørn

**Affiliations:** 1 Center for International Health, University of Bergen, Bergen, Norway; 2 Aklilu Lemma Institute of Pathobiology, Addis Ababa University, Addis Ababa, Ethiopia

## Abstract

**Background:**

Malaria is a public health problem in Ethiopia, and increasingly so in highland areas, possibly because of global warming. This study describes the distribution, breeding habitat and monthly dynamics of anopheline larvae in Butajira, a highland area in south-central Ethiopia.

**Methods:**

A study of the abundance and dynamics of *Anopheles* larvae was undertaken at different sites and altitudes in Butajira from July 2008 to June 2010. The sites included Hobe (1817 m.a.s.l), Dirama (1995m.a.s.l.) and Wurib (2196m.a.s.l.). Potential anopheline larval habitats were surveyed once per month in each village. The recorded characteristics of the habitats included habitat type, pH, surface debris, emergent plants, algae, substrate, turbidity, temperature, length, width, depth, distance to the nearest house and anophelines. The Spearman correlation coefficient and Mann–Whitney *U* test were used to calculate the degree of association between the density of anopheline species and key environmental factors.

**Results:**

Among the different types of habitat surveyed, the Odamo, Akamuja and Assas streams and Beko swamp were positive for anopheline larvae. A total of 3,957 third and fourth instar larvae were collected from the three localities, and they represented ten species of anophelines. These were: *Anopheles cinereus* (32.5%), *An. arabiensis* (31.4%), *An. chrysti* (23%), *An. demeilloni* (12.2%), *An. pretoriensis* (0.6%), *An. azaniae* (0.1%), *An. rufipes*(0.1%), *An. sergentii* (0.06%), *An. garnhami* (0.06%) and *An. pharoensis* (0.03%). The density of anopheline larvae was highest during the dry months. *An. arabiensis* was widely distributed, and its density decreased from the lowest elevation in Hobe to the highest in Wurib. The density of *An. arabiensis* larvae was correlated positively with larval habitat temperature (r = 0.33, *p* < 0.05) and negatively with depth of larval habitat (r = −0.56, *p* < 0.05).

**Conclusion:**

Ten species of anophelines were identified, including two known vectors of malaria (*An. arabiensis* and *An. pharoensis*), along streams in Butajira. Larvae of *An. arabiensis* were found in streams at 2200m.a.s.l. This possible expansion of the malaria vector to highland areas indicates an increasing risk of malaria because a large proportion of the Ethiopian population live above this altitude.

## Background

Malaria is the leading cause of mortality and morbidity in Ethiopia in areas up to 2500 metres above sea level (m.a.s.l) [1], although cases have been reported up to 3000m.a.s.l. in some areas [2]. About 70% of the population is estimated to be at risk of infection every year [3]. Transmission of the disease is unstable in many highland areas of the country, where the population has low immunity, and these regions experience malaria epidemics [4]. *Anopheles arabiensis*, a member of the *An. gambiae* complex, is the main vector of malaria in the country [5], while *An. pharoensisAn. funestus* and *An. nili* represent secondary vectors [6,7].

The transmission of malaria in high altitude areas of Ethiopia [1,2,8] might possibly be due to global warming [9], land use practices [10,11] and ecological changes [12,13] that could favour the breeding and survival of vectors. Warmer weather and increased water temperature enhance malaria transmission in the highlands by shortening the development time from eggs to adult mosquitoes [14], increasing the number of human blood meals taken by adults, increasing the frequency of egg laying and increasing the survival rate of adult mosquitoes [14,15]. Increased warmth also shortens the sporogonic cycle of the parasite in the vector, which results in increased intensity of malaria transmission [16,17]. The continuation of global climate change could therefore allow malaria to expand into the highlands of east Africa [18], threatening the lives of millions of people.

The existing malaria intervention strategy, which includes indoor residual insecticide spraying, nets treated with long-lasting insecticide, and case management, has been reducing the impact of the disease in Ethiopia. Nevertheless, spread of insecticide-resistant vectors [19,20] and drug-resistant malaria parasites [21,22] may result in disease outbreaks. Therefore, control of larvae, which has so far been given little attention, should be reintroduced and implemented together with the existing strategy. Larval control will result in the reduction of the adult mosquito population, subsequently limiting malaria transmission [23]. However, current knowledge of the distribution and dynamics of the aquatic stages of mosquitoes is not adequate. *Anopheles* mosquitoes breed at the edges of rivers and streams, in temporary rain pools, ponds, dams, drainage ditches, burrow pits, rice fields, swamp margins, roadside puddles and in tree holes close to human dwellings [23-25]. However, mosquitoes differ in their preference for the type, size, turbidity, algal cover and stability of the habitat [26-28]; these factors determine the density, size and disease transmission competence of vectors [25,29]. Although malaria has become an important health problem in the south-central highland area of Butajira [30,31], information on the dynamics of the immature stages of the vectors is scarce. The aim of this study was to describe the species distribution and seasonal dynamics of anopheline larvae in the south-central highland area of Butajira. Such information is important in order to implement effective interventions and establish an early warning system for the disease in this country.

## Methods

### Study area

**Figure 1:**
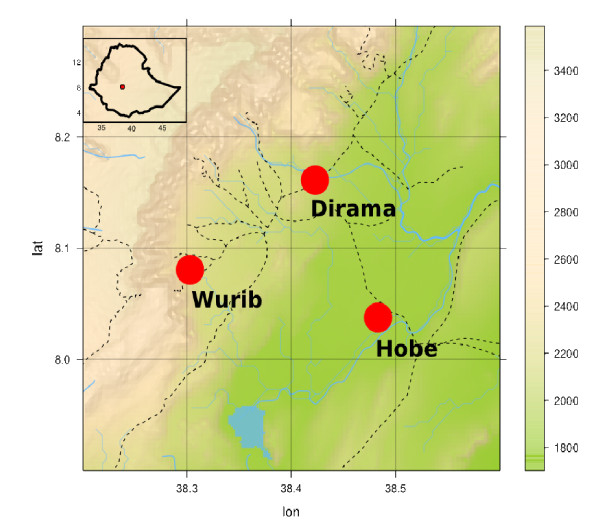
Location of the Butajira study area in the south-central Ethiopian Highlands.

The study was undertaken in the Butajira area in the south-central highlands of the Southern Nations and Nationalities Regional State of Ethiopia, which is located 135 km south of Addis Ababa (Figure 1).

For larval sampling, three study sites were selected. These included Hobe (1817 metres above sea level), Dirama (1995m.a.s.l.) and Wurib (2196 m.a.s.l.). The sites are villages close to the Odamo, Akamuja and Assas streams, respectively. They were selected by Health Extension Workers on the basis of habitat availability, accessibility and malaria case reports. Rainfall data for the area (July 2008 to March 2010) was obtained from the National Meteorological Agency of Ethiopia from the only station in Butajira town, which is located 5–20 km from the study areas. On the basis of the previous thirty years of meteorological data from the area (National Meteorological Agency of Ethiopia), the average monthly rainfall is 94.6 mm and the relative humidity is 60.8%, while the average maximum and minimum temperatures are 25.5°C and 11.5°C, respectively. Peak rainfall occurs between July and August, while the lowest level occurs in November and December, with little rain between March and May.

### Larval sampling and processing

Sampling for anopheline larvae was undertaken once a month from July 2008 to June 2010. Streams, water wells, small rain pools, pools in hoof- or foot-prints and false banana (*Ensete ventricular*) leaf axils were surveyed for the presence of larvae, and collections were made by applying a standard sampling procedure [27,32-34]. Three to ten samples were taken with a soup ladle (350 ml capacity) from each breeding habitat, depending on the size of the habitat and the availability of larvae. In streams, dipping was performed at the edges and stream beds for a distance of 600 to 1600 m, depending on presence of larvae. Along the streams the average distance between two consecutive larval sampling points was 100 m.

Larvae were sorted into culicines and anophelines. All anopheline larvae sampled from each sampling point were identified as 1^st^, 2^nd^, 3^rd^ or 4^th^ larval instars, and the corresponding counts were recorded after transferring the larvae from the sampling dipper to white enamel trays. All culicine larvae and the 1^st^ and 2^nd^ anopheline larval instars were discarded. All late anopheline instars (3^rd^ and 4^th^) were preserved in 70% alcohol after being killed in hot water (ca. 60°C) [35]. In the laboratory, the larvae were mounted in gum-chloral mountant on slides and the species identified on the basis of morphology under a microscope [36]. Furthermore, about 10% of the larval species identified morphologically by the first author (AA) were selected randomly and subjected to reidentification and confirmation by one of the senior and more experienced co-authors (MB). Larvae that were members of the *An. gambiae* complex were inferred from the results of species-specific PCR conducted on the adults collected from the same study sites (manuscript under preparation). After identification of the late instars, the density of the most common species was expressed as the number of larvae per 100 dips.

### Characterization of larval habitat

The types of larval habitats and their characteristics, such as speed, length, width, depth, pH, turbidity, trees nearby (shade), distance to the nearest inhabited house, availability of emergent plants and substrate types were described by technicians and the first author (AA). The flow speed of aquatic habitat was described visually as fast flowing, slowly flowing or stagnant (not flowing). Habitat length, width, depth and distance to the nearest house was measured using measuring tape; shade was recorded as present or absent by observing terrestrial vegetation and/or trees and their branches near the breeding habitat. Emergent plants included both aquatic and immersed terrestrial vegetation [27]. Turbidity was measured by placing a water sample in a clean glass test tube and holding it against a white background; it was classified into four levels: clear, low, medium and high [27]. Substrate type was classified as muddy or sandy. The pH of the water was measured using a portable pH meter, and the water temperature was measured using a minitherm HI 8753 (Romania) digital thermometer.

### Statistical analysis

The data were entered and analysed using SPSS version 16.0 statistical software (SPSS Inc., Chicago, IL). Monthly dynamics of the density of the major anopheline species and the corresponding monthly rainfall data are presented in line charts. The association of the density of the major species with habitat characteristics such as temperature, depth and pH was analysed using the Spearman correlation coefficient, while associations with substrate type (muddy or sandy), turbidity (low or medium), surface debris (present or absent), and surface algae (present or absent) were analysed using the Mann–Whitney *U* test. The extended Mantel–Haenszel chi-square test for linear trend was used to investigate the trends in major anopheline density at the Hope, Dirama and Wurib sites. The Kappa value was calculated to study the agreement between the researchers in the identification of larval species.

## Results

Potential anopheline breeding habitats surveyed from July 2008 to June 2010 in Hobe, Dirama and Wurib villages are presented in Table 1. Among the different types of habitat surveyed, three streams (Odamo, Akamuja and Assas) and one swamp (Beko) were found to harbour anopheline larvae. No anopheline larvae were found in water wells, false banana axils, hoof-prints and most temporary rain pools.

**Table 1:** Aquatic habitats surveyed and anopheline larvae collections in Hobe, Dirama and Wurib Kebeles of Butajira area, south-central Ethiopia (July 2008 to June 2010)

**Kebele**	**Study site**	**Habitat Type (n)**	**Anopheline larvae stages (n)**				
			**1**^**st**^	**2**^**nd**^	**3**^**rd**^	**4**^**th**^	**Total**
Hobe	Hobe	Odamo stream(1)	673	671	691	267	2302
		Wells (5)	0	0	0	0	0
		Rain pools (11)	25	12	0	0	37
		Hoof/Foot prints (20)	0	0	0	0	0
Dirama	Dirama	Akamuja stream (1)	942	741	739	503	2925
		Rain pools (3)	13	0	0	0	13
		Hoof/Foot prints (10)	0	0	0	0	0
		False banana axils (2)	0	0	0	0	0
Wurib	Meter	Assas stream (1)	613	432	390	526	1961
		Wells (2)	0	0	0	0	0
		Rain pools (4)	0	0	0	0	0
		Hoof/Foot prints (9)	0	0	0	0	0
		false banana axils (8)	0	0	0	0	0
	Beko	Beko Swamp (1)	905	558	446	385	2294
		Wells (2)	0	0	0	0	0
		Rain pools (2)	0	0	0	0	0
		Hoof/Foot prints (3)	0	0	0	0	0
		false banana axils (2)	0	0	0	0	0
		**Total**	**3171**	**2414**	**2266**	**1681**	**9532**

Numbers in parentheses represent habitats surveyed.

During the study period, 9532 immature anopheline larvae were collected, of which 3171 (33.3%) were 1^st^ instars, 2414 (25.3%) were 2^nd^ instars, 2266 (23.8%) were 3^rd^ instars and 1681 (17.6%) were 4^th^ instars. Among the total sampled, 2302 were from Odamo stream, 37 from a rain pool in Hobe, 1961 from Assas stream, 2294 from Beko swamp, 2925 from Akamuja Stream and 13 from a foot-print in Dirama village.

Of 3947 late (3^rd^ and 4^th^) instar *Anopheles* larvae, 3100 (78.5%) were identified to species level (Table 2). The remaining 847 (21.5%) were either lost or could not be identified because of damage to larval parts during processing, or were not mounted on slides for identification. Ten percent (n = 305) of the morphologically identified larvae were selected randomly and subjected to re-identification by a second researcher. There was very good agreement (Kappa = 0.89, *p* < 0.01) between the researchers in the morphological identification of the anopheline larvae to species level. *Anopheles cinereus* was the dominant species (32.5%), followed by *An. gambiae* s.l. (= *An. arabiensis* in the present work) (31.4%), *An. chrysti* (23%) and *An. demeilloni* (12.2%).

**Table 2:** Species and distribution of anopheline larvae along the four breeding habitats of Butajira area, south-central Ethiopia (July 2008 –June 2010)

Immature anopheline species	**Breeding habitats**				
	Odamo Stream	Akamuja stream	Assas stream	Beko wamp	Total (%)
*Anopheles cinereus*	10	576	235	187	1008 (32.52)
*Anopheles arabiensis*	684	267	3	19	973 (31.39)
*Anopheles chrysti*	13	118	110	471	712 (22.97)
*Anopheles demeilloni*	10	46	186	136	378 (12.19)
*Anopheles pretoriensis*	0	0	0	17	17 (0.55)
*Anopheles azaniae*	0	1	0	3	4 (0.13)
*Anopheles rufipes*	0	0	0	3	3 (0.10)
*Anopheles sergentii*	0	0	0	2	2 (0.06)
*Anopheles garnhami*	0	1	0	1	2 (0.06)
*Anopheles pharoensis*	0	1	0	0	1(0.03)
**Total**	**717**	**1010**	**534**	**839**	**3100 (100)**

Larvae of *An. arabiensis* were found in the four main breeding sites, with the highest density in Hobe (lowest elevation area) and the lowest density in Wurib (highest elevation area). Larval density declined significantly from the lowland to the highland areas (chi-square for linear trend = 1794, *p* < 0.01). On the other hand, the density of *An. cinereus*, *An. chrysti* and *An. demeilloni* increased from Hobe to Wurib. The six other species, *An. pretoriensis*, *An. rufipes*, *An. sergentii*, *An. azaniae*, *An. garnhami* and *An. Pharoensis*, were rare; the first five were sampled from the high altitude village while the last species was obtained from the intermediate altitude.

**Figure 2:**
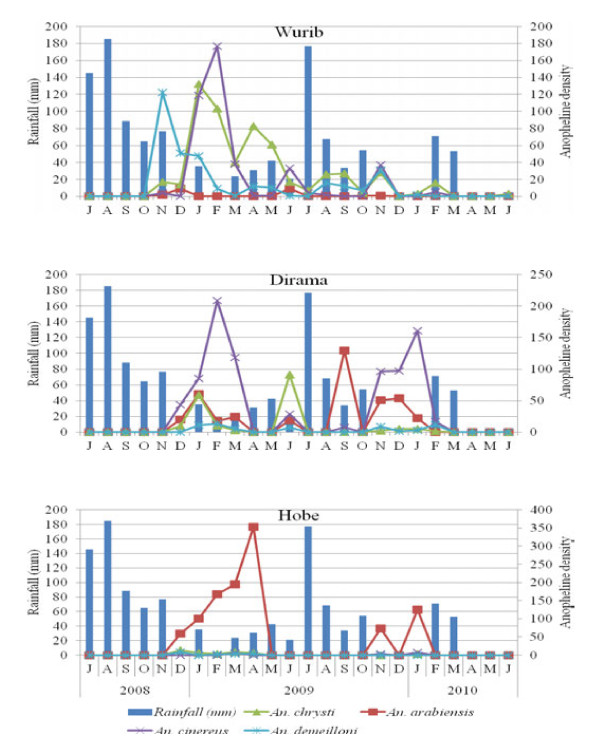
**Monthly rainfall and*Anopheles*larva density in Hobe, Dirama and Wurib villages of Butajira area, south-central Ethiopia, July 2008- June 2010.**

Figure 2 shows the seasonal density of the four common *Anopheles* species, expressed as the number of larvae per 100 ladle dips, and the corresponding monthly rainfall of the area. *An. arabiensis* larvae were predominant in Hobe, with high density from December 2008 to April 2009. This was the dry season, when the monthly rainfall was below 40 mm. The density of *Anopheles* larvae was generally lowest during July and August, corresponding to the highest amount of monthly rainfall. The density of *An. demeilloni*, *An. chrysti* and *An. cinereus* larvae showed similar trends. Among the three villages, Wurib had diverse species of anopheline larvae.

Beko swamp showed the presence of anopheline larvae most frequently (during 16 surveys), followed by Akamuja stream (11 surveys), among the 24 larval surveys (Table 3). The highest average water temperature was recorded along Odamo stream (26°C) and the lowest along Assas stream and in Beko Swamp (23°C). On average, a 1600 m stretch of the Akamuja stream was surveyed once each month for the presence of anophelines, and the shortest habitat distance surveyed, 600 m, was along the Beko swamp. Beko was the deepest permanent breeding habitat (5.3 ± 1.5 cm) and had the closest human inhabitants (20 m).

**Table 3:** Characteristics of streams during anopheline larvae occurrence, south-central Ethiopia, July 2008-June 2010

**Local name of Stream**	**Frequency of occurrence**	**Habitat characteristic (M ± SD)**					
		**Temperature (^0^ C)**	**pH**	**Length (m)**	**Width (m)**	**Depth (cm)**	**Nearest domicile (m)**
Odamo	7	26.1 ± 2.5	7.2 ± 0.3	1597 ± 7.6	4.3 ± 0.5	3.4 ± 0.5	350
Akamuja	11	24.5 ± 1.8	7.5 ± 0.7	1600	5.4 ± 0.5	4.4 ± 0.8	200
Assas	6	22.7 ± 4.1	7.1 ± 0.1	1033 ± 81.7	3.2 ± 0.3	5.0 ± 0.6	120 ± 42.2
Beko Swamp	16	23.0 ± 2.3	7.2 ± 0.3	600	4.4 ± 0.5	5.3 ± 1.5	20

M ± SD = mean ± standard deviation.

The density of *An. arabiensis* late instars increased significantly with increasing habitat temperature (r = 0.33, *p* < 0.01) and also with decreasing depth of habitat (r = −0.56, *p* < 0.05) (Table 4). Analysis using the Mann–Whitney *U* test revealed significantly higher larval density in sandy habitats (z = −3.648, *p* < 0.01) when compared with habitats with muddy substrate. The density of *An. demeilloni* was negatively associated with habitat temperature (r = −0.387, *p* < 0.05). *An. arabiensis*, *An. chrysti*, *An. cinereus* and *An. demeilloni* were not significantly associated with habitat characteristics such as pH, turbidity, surface debris and surface algae in any of the streams. These habitats supported larval development at their shallow edges, where the speed of flow was low, and on their beds in small and stagnant pools. No emergent vegetation was available along the three streams, but was present in Beko swamp. There was no canopy cover along the anopheline-positive habitats, except for some scattered trees, with no measurable shade on the breeding habitats. All the land close to the breeding habitats was cultivated by farmers.

**Table 4:** Association between habitat characteristics and anopheline larval density, south-central Ethiopia, July 2008 to June 2010

	**Species**			
**Habitat characteristics**	*An. arabiensis*	*An. chrysti*	*An. cinereus*	*An. demeilloni*
**Correlation**				
pH	−0.2	0.0	0.1	−0.2
Temperature (^0^ C)	0.3*	−0.1	0.2	−0.3*
Depth (cm)	−0.6**	0.2	0.0	0.2
**Differences of means (medians)**				
Substrate				
Muddy	1.9 (0.0)**	52.5(25.8)*	19.2 (2.3)	15.8 (14.3)
Sandy	61.5 (23.3)	17.7 (4.1)	54.1 (12.5)	16.7 (4.2)
Turbidity				
Low	39.5 (2.5)	31.1 (7.7)	42.2 (7.5)	15.9 (5.0)
Medium	1.1(1.1)	40.9(40.9)	0.0	23.9 (24.0)
Surface debris				
Present	38.0 (2.2)	32.4 (8.7)	42.2 (7.5)	15.0 (5.1)
Absent	30.3 (30.3)	17.7 (17.7)	0.0	42.1 (42.1)
Surface Algae				
Present	38.6 (2.2)	32.2 (8.9)	41.1 (7.4)	16.7 (7.6)
Absent	0.0	8.6 (8.6)	2.9 (2.9)	0.0

* *P* < 0.05; ** *P* < 0.01.

## Discussion

Ten anopheline species were identified in Butajira. The predominant species was *An. Arabiensis*, which is the main vector of malaria in the country [5,6]. Its density decreased from Hobe at the lowest elevation (about 1800 m.a.s.l.) to Wurib at 2200 m.a.s.l. Two of these species (*An. gambiae* s.l, presumably *An. arabiensis* and *An. chrysti*) have been reported from neighbouring villages at about the same altitude [37]. This shows that malaria transmission in the area [30,31] decreases with increasing altitude. Malaria-related mortality in the area was reported previously to follow a similar altitudinal trend [31].

We found that *An. arabiensis* breeds at 2196 m.a.s.l., which is above the altitude reported previously from Kenyan highlands [38,39]. This suggests that malaria vectors are breeding in highland areas, and global warming [9] could be one explanation for the expansion of *An. arabiensis* and *An. pharoensis* in the Butajira highlands. The 1958 malaria epidemic that affected most highland areas, including at 2600 m.a.s.l. [8], and a recent report of malaria prevalence of 3.2% at an altitudinal range of 2500 to 3000 m.a.s.l. [2] could be attributed partly to the expansion of the vectors into areas of higher elevation. Expansion of these vectors to highland areas is a serious threat because most of the Ethiopian population lives in the highlands.

The study revealed that the edges and beds of streams serve as anopheline breeding habitats in the Butajira area during months with low precipitation, as reported previously in the central Rift Valley of Ethiopia [40] and Western Kenya [41]. Streambed pools were also productive breeding habitats of *An. arabiensis* during low rainfall seasons in Eritrea [42]. Similar findings have also been documented in other areas of East Africa [32,42]. Streams can produce large vector populations during dry seasons, and hence larval management that targets streambed pools and stream edges may bring substantial reduction in vector density, and subsequently the incidence of malaria [23,43], in the south-central highlands of Ethiopia. The absence of larvae along the streams during the rainy months could result from increased stream flow, which carries away immature stages of mosquitoes from their breeding points. Heavy rainfall could also kill larvae directly [44].

Larvae could not be investigated in temporary water collections formed during rain, in water wells, or in *Ensete* leaf axils. The absence of larvae from most of the temporary collections of surface water could be due to rapid infiltration of the rain water into the soil and high evaporation. Many permanent water wells did not support anopheline larvae, except culicines. This could be due to their depth, which ranges from 15 to 20 m from the surface, and their water volume, which prevents the entry of direct sunlight and could in turn lower habitat temperature and reduce the availability of the food necessary for larval development. Although temporary habitats may dry out or be flushed out before immature anophelines complete their development [28], they are unpredictable in occurrence and may make a small contribution to overall adult productivity [43]. In addition, their contribution to vector breeding should not be ignored [13] because some may support anopheline breeding. Given that we were not able to perform weekly or daily sampling of larvae, we might have missed some potential and temporary breeding habitats between the monthly surveys, and this could have biased our results. We recommend that future studies should be carried out at frequent intervals to produce more detailed information on the dynamics of anopheline larvae.

The anopheline breeding points were shallow edges and beds of streams that were sunlit, slow flowing or stagnant, with or without debris and surface algae. Similar habitat types were reported from the Ethiopian Rift Valley [40] and Eritrea [25,42]. The larval density of *An. arabiensis* increased with increasing habitat temperature and decreasing habitat depth. The occurrence of *An. arabiensis* larvae in Beko Swamp is an indication of its adaptation to habitats with emergent grass and its expansion to higher elevations, which results in an increased risk of highland malaria. Variability in the pH, turbidity, surface debris and surface algae of the streams did not affect the density of *An. arabiensisAn. chrystiAn. cinereus* and *An. demeilloni* larvae significantly. *An. arabiensis* is adapted to diverse habitats [25,27]. The density of *An. chrystiAn. demeilloni* and *An. cinereus* was not significantly correlated with habitat temperature and depth, which indicates that these anophelines can breed at a greater range of depths and temperatures than *An. arabiensis*. The lower density of the vector (*An. arabiensis*) in the Beko and Assas habitats of Wurib village may have been due to the relatively low temperature in the area, which may affect its breeding negatively. However, this area supported more of other anopheline species for much of the study period, when compared with the other three permanent breeding habitats. This may be because the grass present in this habitat might have prevented the loss of immature forms in running water or by the direct splashing of rainfall, and the grass might have served as a resting site for newly emerging and gravid anopheline mosquitoes [41].

## Conclusion

This study has revealed that *An. arabiensis* breeds on the edges and beds of streams in south-central Ethiopia at elevations up to 2200 m.a.s.l. during the dry months. This observation underlines the importance of streams as breeding habitats of *An. arabiensis* during dry periods. The edges and pools of streams may be important for maintenance of the *Anopheles* population and for small-scale transmission of malaria during dry seasons. Hence, policy makers and organizations involved in malaria control activities need to consider options for the management of larvae that target streams during dry seasons. This strategy may reduce *An. arabiensis* density, and thus reduce the risk of malaria transmission [23,32,42]. However, streams might not be the only breeding habitats for anophelines in the area, and hence weekly surveys of all the available habitats and habitat chemistry need to be performed to design a comprehensive and effective larval control strategy.

## Competing interests

The authors declare that they have no competing interests.

## Authors’ contributions

AA designed the study, collected data in the field, carried out the data analysis and wrote the first draft of the manuscript. TGM participated in the study design, interpretation of the results and editing of the manuscript. MB participated in the conception of the study, in the study design and editing of the manuscript. BL conceived the idea for the study and took part in the study design, data entry and analysis, data interpretation and editing the manuscript. All authors have read and approved the final manuscript.
